# (*E*)-*N*′-(2,4,6-Trimethyl­benzyl­idene)isonicotinohydrazide

**DOI:** 10.1107/S1600536810019446

**Published:** 2010-05-29

**Authors:** H. S. Naveenkumar, Amirin Sadikun, Pazilah Ibrahim, Jia Hao Goh, Hoong-Kun Fun

**Affiliations:** a School of Pharmaceutical Sciences, Universiti Sains Malaysia, 11800 USM, Penang, Malaysia; b X-ray Crystallography Unit, School of Physics, Universiti Sains Malaysia, 11800 USM, Penang, Malaysia

## Abstract

The title isoniazid derivative, C_16_H_17_N_3_O, exists in an *E* configuration with respect to the Schiff base C=N bond. The pyridine ring is essentially planar [maximum deviation = 0.009 (3) Å]. The mean plane through the hydrazide unit forms dihedral angles of 38.38 (16) and 39.42 (16)°, respectively, with the pyridine and benzene rings. In the crystal structure, symmetry-related mol­ecules are linked *via* inter­molecular N—H⋯O hydrogen bonds into chains along [100]. The crystal structure is further stabilized by weak inter­molecular C—H⋯π inter­actions.

## Related literature

For general background to and applications of isoniazid derivatives, see: Janin (2007 ▶); Maccari *et al.* (2005 ▶); Slayden & Barry (2000 ▶); Kahwa *et al.* (1986 ▶). For the preparation of the title compound, see: Lourenco *et al.* (2008 ▶). For related structures, see: Naveenkumar *et al.* (2009 ▶, 2010**a* ▶,b* ▶); Shi (2005 ▶).
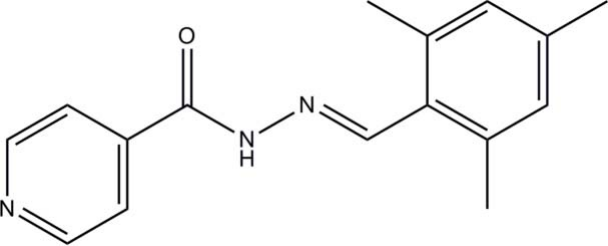


## Experimental

### 

#### Crystal data

C_16_H_17_N_3_O
*M* *_r_* = 267.33
Monoclinic, 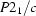

*a* = 4.7966 (7) Å
*b* = 34.268 (7) Å
*c* = 8.3795 (14) Å
β = 96.203 (14)°
*V* = 1369.3 (4) Å^3^
*Z* = 4
Mo *K*α radiation
μ = 0.08 mm^−1^
*T* = 100 K
0.35 × 0.10 × 0.07 mm

#### Data collection

Bruker APEXII DUO CCD area-detector diffractometer
Absorption correction: multi-scan (*SADABS*; Bruker, 2009 ▶) *T*_min_ = 0.971, *T*_max_ = 0.994
12980 measured reflections
3127 independent reflections
2043 reflections with *I* > 2σ(*I*)
*R*_int_ = 0.070

#### Refinement

*R*[*F* ^2^ > 2σ(*F* ^2^)] = 0.074
*wR*(*F* ^2^) = 0.160
*S* = 1.11
3127 reflections
188 parameters
H atoms treated by a mixture of independent and constrained refinement
Δρ_max_ = 0.48 e Å^−3^
Δρ_min_ = −0.30 e Å^−3^

### 

Data collection: *APEX2* (Bruker, 2009 ▶); cell refinement: *SAINT* (Bruker, 2009 ▶); data reduction: *SAINT*; program(s) used to solve structure: *SHELXTL* (Sheldrick, 2008 ▶); program(s) used to refine structure: *SHELXTL*; molecular graphics: *SHELXTL*; software used to prepare material for publication: *SHELXTL* and *PLATON* (Spek, 2009 ▶).

## Supplementary Material

Crystal structure: contains datablocks global, I. DOI: 10.1107/S1600536810019446/lh5051sup1.cif

Structure factors: contains datablocks I. DOI: 10.1107/S1600536810019446/lh5051Isup2.hkl

Additional supplementary materials:  crystallographic information; 3D view; checkCIF report

## Figures and Tables

**Table 1:** Hydrogen-bond geometry (Å, °) *Cg*1 is the centroid of the C1-C5/N1 pyridine ring.

*D*—H⋯*A*	*D*—H	H⋯*A*	*D*⋯*A*	*D*—H⋯*A*
N2—H1*N*2⋯O1^i^	0.93 (3)	1.97 (3)	2.844 (3)	157 (3)
C16—H16*B*⋯*Cg*1^i^	0.96	2.96	3.551 (3)	121

Symmetry code: (i) 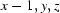.

**Fig. 1.**
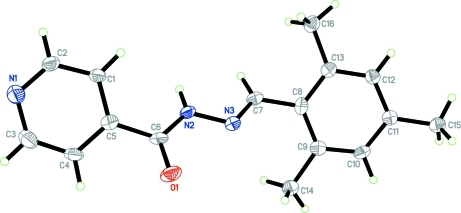
The molecular structure of the title derivative with atom labels and 50% probability ellipsoids for non-H atoms.

**Fig. 2.**
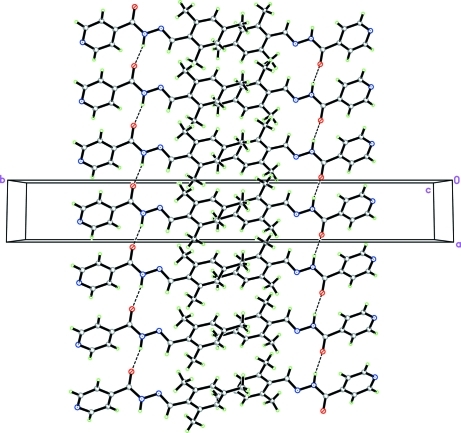
The crystal structure of title derivative, viewed along the c axis, showing adjacent molecules being linked into one-dimensional chains along the [100] direction. Intermolecular hydrogen bonds are shown as dashed lines.

## supplementary crystallographic information

### Comment

In the search of new compounds, isoniazid derivatives have been found to possess potential tuberculostatic activity (Janin, 2007; Maccari *et al.*, 2005; Slayden & Barry, 2000). Schiff bases have attracted much attention because of their biological activity (Kahwa *et al.*, 1986). As part of our current work on the synthesis of (*E*)-*N*'-substituted isonicotinohydrazide derivatives, in this paper we report the crystal structure of the title isoniazid derivative.

The title isoniazid derivative (Fig. 1) exists in an *E* configuration with respective to the Schiff base C7═N3 bond [C7═N3 = 1.280 (3) Å; torsion angle N2–N3–C7–C8 = 179.4 (2)°]. The pyridine ring with atom sequence C1/C2/N1/C3/C4/C5 is essentially planar, with a maximum deviation of 0.009 (3) Å at atom C4. The mean plane through the hydrazide unit (O1/C6/N2/N3/C7) forms dihedral angles of 38.38 (16) and 39.42 (16)°, respectively, with the pyridine and benzene (C8-C13) rings. The bond lengths and angles are consistent to those observed in closely related structures (Naveenkumar *et al.*, 2009; 2010*a,b*; Shi, 2005).

In the crystal structure (Fig. 2), adjacent molecules are linked into one-dimensional chains along the [100] direction *via* intermolecular N2—H1N2···O1^i^ hydrogen bonds (Table 1). The crystal structure is further stabilized by weak intermolecular C16—H16B···*Cg*1^i^ interactions (Table 1) involving the centroid of the pyridine ring.

### Experimental

The title isoniazid derivative was prepared following the procedure by Lourenco *et al.*, (2008). The title derivative was prepared by the reaction between 2,4,6-trimethylbenzaldehyde (1.0 eq) with isoniazid (1.0 eq) in ethanol/water. After stirring for 1-3 h at room temperature, the resulting mixture was concentrated under reduced pressure. The residue was purified by washing with cold ethanol and ethyl ether to afford the pure derivative. Colourless single crystals suitable for X-ray analysis were obtained by slow evaporation with dimethyl sulfoxide.

### Refinement

Atom H1N2 was located from difference Fourier map and allowed to refine freely. All other H atoms were placed in calculated positions, with C—H = 0.93 or 0.96 Å, *U*_iso_ = 1.2 or 1.5 *U*_eq_(C). These H atoms were refined as riding on their parent atoms. A rotating group model was used for the methyl groups.

### Crystal data

**Table:**

C_16_H_17_N_3_O	*F*(000) = 568
*M**_r_* = 267.33	*D*_x_ = 1.297 Mg m^−^^3^
Monoclinic, *P*2_1_/*c*	Mo *K*α radiation, λ = 0.71073 Å
Hall symbol: -P 2ybc	Cell parameters from 2541 reflections
*a* = 4.7966 (7) Å	θ = 2.4–30.0°
*b* = 34.268 (7) Å	µ = 0.08 mm^−^^1^
*c* = 8.3795 (14) Å	*T* = 100 K
β = 96.203 (14)°	Needle, colourless
*V* = 1369.3 (4) Å^3^	0.35 × 0.10 × 0.07 mm
*Z* = 4	

### Data collection

**Table:**

Bruker APEXII DUO CCD area-detector diffractometer	3127 independent reflections
Radiation source: fine-focus sealed tube	2043 reflections with *I* > 2σ(*I*)
graphite	*R*_int_ = 0.070
φ and ω scans	θ_max_ = 27.5°, θ_min_ = 2.4°
Absorption correction: multi-scan (*SADABS*; Bruker, 2009)	*h* = −6→6
*T*_min_ = 0.971, *T*_max_ = 0.994	*k* = −44→43
12980 measured reflections	*l* = −10→10

### Refinement

**Table:**

Refinement on *F*^2^	Primary atom site location: structure-invariant direct methods
Least-squares matrix: full	Secondary atom site location: difference Fourier map
*R*[*F*^2^ > 2σ(*F*^2^)] = 0.074	Hydrogen site location: inferred from neighbouring sites
*wR*(*F*^2^) = 0.160	H atoms treated by a mixture of independent and constrained refinement
*S* = 1.11	*w* = 1/[σ^2^(*F*_o_^2^) + (0.0432*P*)^2^ + 1.4572*P*] where *P* = (*F*_o_^2^ + 2*F*_c_^2^)/3
3127 reflections	(Δ/σ)_max_ < 0.001
188 parameters	Δρ_max_ = 0.48 e Å^−^^3^
0 restraints	Δρ_min_ = −0.30 e Å^−^^3^

### Special details

**Table:**

Experimental. The crystal was placed in the cold stream of an Oxford Cryosystems Cobra open-flow nitrogen cryostat (Cosier & Glazer, 1986) operating at 100.0 (1)K.
Geometry. All esds (except the esd in the dihedral angle between two l.s. planes) are estimated using the full covariance matrix. The cell esds are taken into account individually in the estimation of esds in distances, angles and torsion angles; correlations between esds in cell parameters are only used when they are defined by crystal symmetry. An approximate (isotropic) treatment of cell esds is used for estimating esds involving l.s. planes.
Refinement. Refinement of F^2^ against ALL reflections. The weighted R-factor wR and goodness of fit S are based on F^2^, conventional R-factors R are based on F, with F set to zero for negative F^2^. The threshold expression of F^2^ > 2sigma(F^2^) is used only for calculating R-factors(gt) etc. and is not relevant to the choice of reflections for refinement. R-factors based on F^2^ are statistically about twice as large as those based on F, and R- factors based on ALL data will be even larger.

### Fractional atomic coordinates and isotropic or equivalent isotropic displacement parameters (Å^2^)

**Table:**

	*x*	*y*	*z*	*U*_iso_*/*U*_eq_	
O1	0.9087 (4)	0.22119 (6)	0.3351 (3)	0.0317 (5)	
N1	0.3263 (5)	0.34311 (7)	0.2703 (3)	0.0306 (6)	
N2	0.4637 (5)	0.19838 (6)	0.2872 (3)	0.0213 (5)	
N3	0.5529 (4)	0.16006 (6)	0.2958 (3)	0.0225 (5)	
C1	0.3140 (5)	0.27683 (7)	0.1801 (3)	0.0219 (6)	
H1A	0.2303	0.2581	0.1102	0.026*	
C2	0.2194 (6)	0.31475 (8)	0.1741 (4)	0.0258 (6)	
H2A	0.0708	0.3210	0.0977	0.031*	
C3	0.5418 (6)	0.33331 (8)	0.3769 (4)	0.0329 (7)	
H3A	0.6210	0.3526	0.4457	0.039*	
C4	0.6534 (6)	0.29642 (8)	0.3909 (3)	0.0268 (6)	
H4A	0.8062	0.2913	0.4660	0.032*	
C5	0.5375 (5)	0.26708 (7)	0.2929 (3)	0.0213 (6)	
C6	0.6561 (5)	0.22694 (8)	0.3077 (3)	0.0216 (6)	
C7	0.3557 (5)	0.13476 (7)	0.2787 (3)	0.0225 (6)	
H7A	0.1699	0.1429	0.2631	0.027*	
C8	0.4207 (5)	0.09301 (7)	0.2837 (3)	0.0218 (6)	
C9	0.6338 (5)	0.07743 (8)	0.3932 (3)	0.0230 (6)	
C10	0.6948 (5)	0.03831 (8)	0.3826 (3)	0.0239 (6)	
H10A	0.8364	0.0279	0.4547	0.029*	
C11	0.5558 (5)	0.01384 (7)	0.2701 (3)	0.0219 (6)	
C12	0.3405 (5)	0.02938 (7)	0.1670 (3)	0.0219 (6)	
H12A	0.2411	0.0131	0.0924	0.026*	
C13	0.2675 (5)	0.06847 (7)	0.1712 (3)	0.0212 (6)	
C14	0.7896 (6)	0.10107 (8)	0.5253 (3)	0.0289 (7)	
H14A	0.8591	0.0841	0.6117	0.043*	
H14B	0.6652	0.1200	0.5639	0.043*	
H14C	0.9440	0.1142	0.4848	0.043*	
C15	0.6406 (6)	−0.02825 (8)	0.2617 (3)	0.0278 (6)	
H15A	0.6317	−0.0403	0.3644	0.042*	
H15C	0.8287	−0.0299	0.2331	0.042*	
H15D	0.5155	−0.0415	0.1823	0.042*	
C16	0.0339 (5)	0.08385 (8)	0.0537 (3)	0.0266 (6)	
H16A	−0.0566	0.0625	−0.0053	0.040*	
H16D	0.1094	0.1015	−0.0194	0.040*	
H16B	−0.1001	0.0973	0.1108	0.040*	
H1N2	0.271 (7)	0.2019 (8)	0.278 (4)	0.033 (8)*	

### Atomic displacement parameters (Å^2^)

**Table:**

	*U*^11^	*U*^22^	*U*^33^	*U*^12^	*U*^13^	*U*^23^
O1	0.0158 (10)	0.0349 (11)	0.0431 (13)	0.0011 (8)	−0.0035 (9)	0.0082 (9)
N1	0.0296 (14)	0.0280 (13)	0.0340 (15)	0.0002 (10)	0.0018 (11)	−0.0029 (11)
N2	0.0157 (12)	0.0217 (11)	0.0252 (13)	0.0021 (8)	−0.0035 (9)	0.0027 (9)
N3	0.0204 (12)	0.0230 (12)	0.0232 (13)	0.0026 (9)	−0.0012 (9)	0.0003 (9)
C1	0.0199 (13)	0.0243 (14)	0.0207 (15)	−0.0034 (10)	−0.0023 (11)	0.0017 (11)
C2	0.0199 (14)	0.0277 (15)	0.0287 (16)	0.0012 (11)	−0.0029 (12)	0.0033 (12)
C3	0.0346 (18)	0.0314 (16)	0.0317 (18)	−0.0073 (12)	−0.0013 (14)	−0.0062 (13)
C4	0.0228 (14)	0.0329 (16)	0.0227 (15)	−0.0039 (11)	−0.0068 (12)	0.0000 (12)
C5	0.0172 (13)	0.0267 (14)	0.0206 (14)	−0.0013 (10)	0.0039 (11)	0.0045 (11)
C6	0.0153 (13)	0.0291 (14)	0.0193 (14)	−0.0018 (10)	−0.0033 (11)	0.0048 (11)
C7	0.0163 (13)	0.0282 (14)	0.0226 (15)	0.0023 (10)	0.0007 (11)	0.0024 (11)
C8	0.0244 (14)	0.0175 (13)	0.0252 (15)	0.0002 (10)	0.0103 (12)	0.0013 (11)
C9	0.0224 (14)	0.0269 (14)	0.0200 (15)	−0.0015 (11)	0.0031 (11)	0.0018 (11)
C10	0.0203 (14)	0.0291 (15)	0.0213 (15)	0.0046 (10)	−0.0019 (11)	0.0046 (11)
C11	0.0211 (14)	0.0245 (14)	0.0205 (14)	−0.0005 (10)	0.0037 (11)	0.0016 (11)
C12	0.0206 (14)	0.0222 (13)	0.0231 (15)	−0.0016 (10)	0.0032 (11)	−0.0006 (11)
C13	0.0161 (13)	0.0243 (14)	0.0239 (15)	−0.0005 (10)	0.0059 (11)	0.0028 (11)
C14	0.0318 (16)	0.0275 (15)	0.0261 (16)	0.0015 (11)	−0.0028 (13)	0.0014 (12)
C15	0.0316 (16)	0.0277 (15)	0.0232 (16)	0.0053 (12)	−0.0018 (12)	0.0022 (12)
C16	0.0214 (14)	0.0246 (14)	0.0337 (17)	0.0007 (11)	0.0033 (12)	0.0015 (12)

### Geometric parameters (Å, °)

**Table:**

O1—C6	1.225 (3)	C8—C13	1.409 (4)
N1—C2	1.330 (4)	C9—C10	1.377 (4)
N1—C3	1.334 (4)	C9—C14	1.504 (4)
N2—C6	1.343 (3)	C10—C11	1.378 (4)
N2—N3	1.380 (3)	C10—H10A	0.9300
N2—H1N2	0.93 (3)	C11—C12	1.380 (4)
N3—C7	1.280 (3)	C11—C15	1.502 (4)
C1—C2	1.375 (4)	C12—C13	1.386 (3)
C1—C5	1.391 (4)	C12—H12A	0.9300
C1—H1A	0.9300	C13—C16	1.505 (4)
C2—H2A	0.9300	C14—H14A	0.9600
C3—C4	1.373 (4)	C14—H14B	0.9600
C3—H3A	0.9300	C14—H14C	0.9600
C4—C5	1.377 (4)	C15—H15A	0.9600
C4—H4A	0.9300	C15—H15C	0.9600
C5—C6	1.489 (4)	C15—H15D	0.9600
C7—C8	1.464 (4)	C16—H16A	0.9600
C7—H7A	0.9300	C16—H16D	0.9600
C8—C9	1.403 (4)	C16—H16B	0.9600
			
C2—N1—C3	116.2 (2)	C8—C9—C14	123.1 (2)
C6—N2—N3	118.8 (2)	C9—C10—C11	123.1 (3)
C6—N2—H1N2	125.5 (18)	C9—C10—H10A	118.5
N3—N2—H1N2	115.4 (18)	C11—C10—H10A	118.5
C7—N3—N2	114.7 (2)	C10—C11—C12	117.9 (2)
C2—C1—C5	118.6 (3)	C10—C11—C15	120.2 (2)
C2—C1—H1A	120.7	C12—C11—C15	122.0 (2)
C5—C1—H1A	120.7	C11—C12—C13	122.1 (3)
N1—C2—C1	124.4 (3)	C11—C12—H12A	119.0
N1—C2—H2A	117.8	C13—C12—H12A	119.0
C1—C2—H2A	117.8	C12—C13—C8	118.6 (2)
N1—C3—C4	123.8 (3)	C12—C13—C16	119.6 (2)
N1—C3—H3A	118.1	C8—C13—C16	121.8 (2)
C4—C3—H3A	118.1	C9—C14—H14A	109.5
C3—C4—C5	119.5 (3)	C9—C14—H14B	109.5
C3—C4—H4A	120.2	H14A—C14—H14B	109.5
C5—C4—H4A	120.2	C9—C14—H14C	109.5
C4—C5—C1	117.5 (2)	H14A—C14—H14C	109.5
C4—C5—C6	119.9 (2)	H14B—C14—H14C	109.5
C1—C5—C6	122.5 (2)	C11—C15—H15A	109.5
O1—C6—N2	124.0 (2)	C11—C15—H15C	109.5
O1—C6—C5	121.7 (2)	H15A—C15—H15C	109.5
N2—C6—C5	114.3 (2)	C11—C15—H15D	109.5
N3—C7—C8	120.4 (2)	H15A—C15—H15D	109.5
N3—C7—H7A	119.8	H15C—C15—H15D	109.5
C8—C7—H7A	119.8	C13—C16—H16A	109.5
C9—C8—C13	120.1 (2)	C13—C16—H16D	109.5
C9—C8—C7	121.9 (2)	H16A—C16—H16D	109.5
C13—C8—C7	118.0 (2)	C13—C16—H16B	109.5
C10—C9—C8	118.2 (2)	H16A—C16—H16B	109.5
C10—C9—C14	118.7 (2)	H16D—C16—H16B	109.5
			
C6—N2—N3—C7	178.4 (2)	N3—C7—C8—C13	−138.1 (3)
C3—N1—C2—C1	0.9 (4)	C13—C8—C9—C10	2.7 (4)
C5—C1—C2—N1	−0.5 (4)	C7—C8—C9—C10	−175.6 (2)
C2—N1—C3—C4	−0.1 (4)	C13—C8—C9—C14	−174.7 (2)
N1—C3—C4—C5	−1.2 (5)	C7—C8—C9—C14	7.1 (4)
C3—C4—C5—C1	1.7 (4)	C8—C9—C10—C11	−0.3 (4)
C3—C4—C5—C6	−179.7 (3)	C14—C9—C10—C11	177.2 (2)
C2—C1—C5—C4	−0.9 (4)	C9—C10—C11—C12	−1.9 (4)
C2—C1—C5—C6	−179.5 (2)	C9—C10—C11—C15	177.8 (3)
N3—N2—C6—O1	−0.6 (4)	C10—C11—C12—C13	1.8 (4)
N3—N2—C6—C5	178.9 (2)	C15—C11—C12—C13	−177.9 (2)
C4—C5—C6—O1	−37.5 (4)	C11—C12—C13—C8	0.6 (4)
C1—C5—C6—O1	141.0 (3)	C11—C12—C13—C16	178.9 (2)
C4—C5—C6—N2	142.9 (3)	C9—C8—C13—C12	−2.8 (4)
C1—C5—C6—N2	−38.5 (4)	C7—C8—C13—C12	175.5 (2)
N2—N3—C7—C8	179.4 (2)	C9—C8—C13—C16	178.9 (2)
N3—C7—C8—C9	40.1 (4)	C7—C8—C13—C16	−2.9 (4)

### Hydrogen-bond geometry (Å, °)

**Table:**

Cg1 is the centroid of the C1-C5/N1 pyridine ring.

**Table:**

*D*—H···*A*	*D*—H	H···*A*	*D*···*A*	*D*—H···*A*
N2—H1N2···O1^i^	0.93 (3)	1.97 (3)	2.844 (3)	157 (3)
C16—H16B···Cg1^i^	0.96	2.96	3.551 (3)	121

Symmetry codes: (i) *x*−1, *y*, *z*.
